# How to Gargle

**Published:** 1900-02-03

**Authors:** 


### HOW TO GARGLE.

A writer in the *Charlotte Medical Journal* points out that if one is to really do good by gargling—that is, if one is to ensure that the fluid shall reach the posterior wall of the pharynx—the nose must be held and the head must be well thrown back while performing the gargling process. He says that by gargling in the usual way only the anterior surface of the uvula and soft palate and the base of the tongue are reached. But by holding the nose and throwing the head well back when gargling the medicament reaches every surface of the pharynx very effectively. The comparative value of the two methods can be tested by painting the posterior surface of the pharynx carefully with a strong solution of methylene blue, and then letting the patient gargle with water in the usual way when it will be found that the water ejected will be clear and un[illegible]ned; then let him gargle again, holding the nose and throwing his head well back, when th ejected fluid will be found stained, and an inspection o the pharynx will show that the blue has been washed away. This is a thing worth remembering, for many observers have maintained that gargling is not only useless as a method of medication, but is quite in effectual even as a means of cleansing the pharynx.

